# SARS‐CoV‐2 nucleocapsid protein interacts with immunoregulators and stress granules and phase separates to form liquid droplets

**DOI:** 10.1002/1873-3468.14229

**Published:** 2021-11-22

**Authors:** Syam Prakash Somasekharan, Martin Gleave

**Affiliations:** ^1^ Department of Urologic Sciences Faculty of Medicine Vancouver Prostate Centre University of British Columbia Vancouver BC Canada

**Keywords:** G3BP1, liquid–liquid phase separation, mitoxantrone, NCAP, nucleocapsid, SARS‐CoV‐2, SILAC, stress granules, viral factory, viral infection

## Abstract

The current work investigated SARS‐CoV‐2 Nucleocapsid (NCAP or N protein) interactors in A549 human lung cancer cells using a SILAC‐based mass spectrometry approach. NCAP interactors included proteins of the stress granule (SG) machinery and immunoregulators. NCAP showed specific interaction with the SG proteins G3BP1, G3BP2, YTHDF3, USP10 and PKR, and translocated to SGs following oxidative stress and heat shock. Treatment of recombinant NCAP with RNA isolated from A549 cells exposed to oxidative stress‐stimulated NCAP to undergo liquid–liquid phase separation (LLPS). RNA degradation using RNase A treatment completely blocked the LLPS property of NCAP as well as its SG association. The RNA intercalator mitoxantrone also disrupted NCAP assembly *in vitro* and in cells. This study provides insight into the biological processes and biophysical properties of the SARS‐CoV‐2 NCAP.

## Graphical abstract

We have identified novel interactors of the SARS‐CoV‐2 nucleocapsid (NCAP) protein in human lung epithelial cancer cells. NCAP showed specific interaction with the stress granule (SG) proteins G3BP1, G3BP2, YTHDF3, USP10 and PKR, and translocated to SGs following stress treatments. NCAP exhibited RNA‐dependent phase separation. The RNA intercalator mitoxantrone disrupted NCAP assembly *in vitro* and in cells. This study provides insight into the biological processes and biophysical properties of the NCAP.

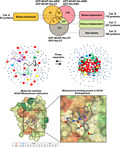

## Abbreviations

**LLPS**, liquid–liquid phase separation

**mRNP**, messenger ribonucleoprotein

**NBs**, Negri bodies

**NCAP**, nucleocapsid

**RBPs**, RNA‐binding proteins

**SGs**, stress granules

**SILAC**, stable‐isotope labelling by amino acids in cell culture

**VFs**, viral factories

Severe acute respiratory syndrome coronavirus 2 (SARS‐CoV‐2) causes coronavirus disease (COVID‐19). The virus primarily affects respiratory tracts, demonstrates rapid human‐to‐human transmission, and spread quickly to different world locations [1, 2, 3]. Although multiple attempts are underway to contain this pandemic, there is no specific targeted therapeutic yet. In addition, limited knowledge hampers the development of antivirals against SARS‐CoV‐2. Therefore, a better understanding of the virus’s biology and mechanisms of hijacking its host translation and immune systems can provide critical insights for developing potent antiviral therapeutics.

SARS‐CoV‐2 contains 30 000 nucleotides single‐stranded RNA genome [4]. The first two‐thirds of the genome translates into two polyproteins (pp1a and pp1ab) and encodes most non‐structural proteins (nsp). The remaining regions encode accessory proteins and four essential structural proteins – spike (S), envelope (E), membrane (M) and nucleocapsid (N or NCAP). NCAP is a multifunctional RNA‐binding protein containing two distinct N‐terminal (NTD) and C‐terminal (CTD) RNA‐binding domains. A disordered linkage region (LKR) with a serine‐arginine‐rich domain (SRD) links these two domains [5]. NCAP binds and regulates the viral genome [6] and, in general, has a multifunctional role in the viral life cycle and host cell antiviral response. Since NCAP is expressed at higher levels in infected cells and is highly immunogenic, it is a candidate target for vaccine and antiviral drug development [7, 8].

Viruses form viral replication compartments [viral factories (VFs) or viral replication factories (RFs)] using viral and host proteins [9, 10, 11, 12]. RFs are the sites for viral transcription, replication and assembly [13]. In certain cases, RFs support the virus to escape from the host cell immune response. For instance, reovirus inhibits antiviral interferon production by sequestering the interferon regulatory factor 3 (IRF3) into viral factories [14]. In addition to RFs, viruses also induce dynamic cytoplasmic RNA‐protein foci called stress granules (SGs). SGs are triggered by several types of viruses, including reovirus [15, 16], hepatitis C virus [17], poliovirus [18], respiratory syncytial virus [19], mouse hepatitis coronavirus [20] and vaccinia virus [21]. SGs contain translationally silenced messenger ribonucleoprotein (mRNP) condensates which form in response to a wide range of cellular stresses [22]. SGs function as a platform to activate the innate immune response to viral infection. The SG nucleator protein, G3BP1, promotes antiviral activity and is essential in double‐stranded RNA‐dependent protein kinase (PKR) recruitment to SGs, thereby driving the phosphorylation of eIF2α to limit translation of viral proteins [13, 16, 23, 24, 25]. Viruses have also evolved mechanisms that antagonize SG responses as part of the infectious cycle. G3BP1 is often cleaved by virus‐encoded proteases or sequestered in an inactive state by viral proteins to block SGs, which help the virus usurp host machinery to translate viral proteins [26, 27]. Infection with flaviviruses, including West Nile virus and dengue virus, also interfere SG formation and are linked to reduced levels of host mRNAs [28].

RFs and SGs have been shown to be closely connected [13]. RFs can function as a primary location for viral protein synthesis by interacting with cellular translation initiation factors and components of the pre‐initiation ribosomal complexes at the factory margins [29]. In rabies virus, SGs localized in close proximity to viral factories, known as Negri bodies (NBs). The three‐dimensional reconstructions showed that both NBs and SGs remained distinct while they were in close contact. Also, viral mRNAs synthesized in NBs accumulated in the SGs during viral infection, indicating material exchange between the compartments [30]. In vaccinia virus, SG‐like bodies form in proximity to replication factories, promoting translation [31]. Mammalian orthoreovirus infection led to the localization of SG‐associated proteins to the periphery of VFs. This re‐localization was proposed to play roles in SG disruption to facilitate viral replication when the host translational machinery is shutdown [12]. The exact mechanism of protein and transcript compartmentalization between SGs and RFs is not well‐defined. SGs are a highly dynamic and rich source of translation initiation factors and RNA‐binding proteins (RBPs) [32]. Viruses may use this resource to translate viral mRNAs during the viral life cycle. For instance, the assembly and disassembly of SGs can be hijacked and modulated by their association with viral nucleoproteins, identifying SGs as a potential reservoir of components that support the translation of viral mRNAs. Viruses might also use SGs to sequester and protect viral mRNAs from degradation by host cell nucleases activated as part of the host immune response [33, 34]. This study investigated the interactors of NCAP using a SILAC‐based approach in A549 human lung cancer cells. NCAP interacted with many SG and immunoregulatory proteins in unstressed and oxidatively stressed cells. Pathway analysis indicated that NCAP interacting proteins have diverse biological functions. NCAP strongly reduced the translation and assembled in SGs in response to oxidative and heat stress. Biophysical studies using recombinant NCAP revealed the protein’s RNA‐dependent phase separation capacity to form liquid droplets. The chelating drug mitoxantrone disrupted RNA‐dependent NCAP assembly and SG association. This study provides insights into NCAP’s interactors and possible biological functions, modulation of SGs, biophysical characters and demonstrates a strategy to target this protein.

## Materials and methods

### Reagents

DF‐12K medium, l‐glutamine, FBS and trypsin were from Sigma‐Aldrich. Antibody against SARS‐CoV‐2 Nucleocapsid Protein (Cat. No. 26369) and USP10 (Cat. No. 5553S) were from Cell Signaling. Antibodies against TIA1 (Cat. No. ab140595), G3BP1 (Cat. No. ab56574), His‐tag (Cat. No. ab18184), YB‐1 (Cat. No. ab76149) and YTHDF3 (Cat. No. ab220161) were from Abcam. Antibodies against G3BP2 (Cat. No. NBP1‐82976), CAPRIN1 (Cat. No. NBP2‐22238) and DDX6 (Cat. No. NB200‐191) were from Novus Biologicals. Antibody against β‐Actin (Cat. No. sc‐47778) was from Santa Cruz Biotechnology. Fluorescently labelled secondary antibodies (mouse, Alexa Fluor 488/594; rabbit, Alexa Fluor 488/594) and Protein A Magnetic Beads were from Life Technologies. 1,6‐Hexanediol was from Sigma. TransIT‐2020 transfection reagent was from Mirus. HisPur™ Ni‐NTA Spin Purification Kit (1 mL), Zeba Spin desalting columns (7K MWCO 2 mL) and LightShift Chemiluminescent RNA EMSA kit were from Thermo Fisher Scientific.

### Cell culture

A549 lung carcinoma cells were procured from ATCC (ATCC® CCL‐185™). The cells were cultured in DF‐12K medium supplemented with a final concentration of 10% FBS. The cell cultures were maintained at a cell concentration between 6 × 10^3^ and 6 × 10^4^ cell·cm^−2^ at 37 °C with 95% air and 5% carbon dioxide (CO_2_).

### SILAC mass spectrometry

SILAC experiments were conducted as described before [35, 36, 37]. Cells were maintained in culture media without arginine and lysine, and supplemented with light (R0K0) or heavy (R10K8) amino acids, dialysed fetal bovine serum and penicillin/streptomycin. Cells were maintained in SILAC media for a minimum of five cell divisions to achieve > 95% incorporation of labelled amino acids. Mass spectrometry analysis of sample lysates from the labelled cells confirmed the incorporation of heavy amino acids. Four sets of cell culture conditions were established: In the first two sets, cells growing in heavy amino acid media were transfected with the control plasmid (GFP‐His‐Heavy‐UT), and cells growing in light amino acid media were transfected with NCAP plasmid (GFP‐NCAP‐His‐Light‐UT), and these cells were kept as untreated. In the second two sets, the plasmid transfections were reversed, that is, cells growing in light amino acid media were transfected with the control plasmid (GFP‐His‐Light‐ARS), and cells growing in heavy amino acid media were transfected with NCAP plasmid (GFP‐NCAP‐His‐Heavy‐ARS), and these cells were treated with 100 μm arsenite for 1 h. Cell lysates were prepared from all conditions, and an equal amount of proteins were incubated with anti‐His antibodies to pulldown the tagged proteins from respective conditions. The immunoprecipitated samples from different conditions were mixed to generate three samples (GFP‐His‐Heavy‐UT: GFP‐NCAP‐His‐Light‐UT; GFP‐His‐Light‐ARS: GFP‐NCAP‐His‐Heavy‐ARS and GFP‐NCAP‐His‐Light‐UT: GFP‐NCAP‐His‐Heavy‐ARS). The samples were processed using established methods described before [35, 36] and analysed in an Orbitrap Fusion Lumos MS platform (Thermo Scientific, Waltham, MA, USA).

### Expression and purification of proteins

N‐GFP‐NCAP‐His_pET‐28a(+)‐TEV and N‐RFP‐G3BP1‐His_pET‐28a(+)‐TEV were custom synthesized from GenScript. The plasmids were transfected to BL21(DE3) pLysS competent cells (Promega). Positive colonies were picked up and grown at 37 °C in LB media supplemented with 50 μg mL^−1^ of kanamycin until the early exponential phase (OD600 0.4–0.8). Isopropyl‐β‐d‐1‐thiogalactopyranoside (IPTG) was added to the culture to a final concentration of 0.5 mm, and protein expression was induced by further incubating the culture at 37 °C for 5 h. The cells were harvested by centrifugation at 4700  ***g*** for 10 min, resuspended in 10 mL lysis buffer (10 mm imidazole in 1X PBS) and subjected to sonication. The suspension was then cleared by centrifugation at 9600  ***g*** for 30 min at 4 °C temperature, and the resulting supernatant was passed through a 1 mL Ni‐NTA column (Thermo Scientific). After washing the column with washing buffer (50 mm imidazole in PBS), recombinant proteins were eluted with elution buffer (250 mm imidazole in PBS) and dialysed extensively against PBS to remove the imidazole.

### Immunoblotting

Cells were gently scraped off from the culture dishes with a cell scraper, washed with PBS and lysed using lysis buffer (20 mm Tris‐HCl, pH 7.5, 150 mm NaCl, 1 mm Na_2_EDTA, 1 mm EGTA, 1% Triton X‐100 and 1× protease inhibitor). Cell lysates were centrifuged at 2400 ***g*** for 10 min, and the supernatant was saved. Protein concentration was determined using a Bradford assay (Bio‐Rad Laboratories, Hercules, CA, USA). Protein lysates were mixed with 2× loading dye, and an equal amount of proteins were separated in 4–12% gradient SDS/PAGE and immunoblotted into nitrocellulose membrane using wet transfer as described previously [38].

### Fluorescence microscopy

Cells seeded at 20–25% confluence in 6 cm culture dishes containing round cover glasses (12CIR‐1D; Thermo Fisher Scientific) were transfected with GFP‐NCAP and exposed to −/+ arsenite treatment (100 μm) or heat shock (41 °C) for 1 h. RNase A treatment of cells was done as described previously [39]. Briefly, GFP‐NCAP expressing cells were subjected to arsenite or heat shock for 50 min followed by treatment with 100 μg mL^−1^ of RNase A for 10 min in PBS containing 0.01% Triton X‐100 to permeabilize the cell membrane and degrade the RNA in live cells. For the mitoxantrone experiments, GFP‐NCAP expressing cells were untreated or treated with 1 μm mitoxantrone for 6 h before exposing them to 100 μm arsenite or 41 °C heat shock, both for 1 h. The above cells were subjected to IF, as described previously [22]. Cells were fixed in 4% paraformaldehyde (PAF) for 20 min and permeabilized with PBS‐T (0.05% Triton X‐100 in PBS) for 20 min. The cells were then blocked for 30 min in PBS‐T containing 5% BSA and incubated with primary antibodies (1 : 100) for 1 h in PBS containing 2.5% BSA. Cells were washed in PBS‐T for 30 min (3 × 10 min) followed by incubation with secondary antibodies (1 : 200) in PBS‐T containing 2.5% BSA for 1 h. Cells were then washed in PBS‐T for 30 min (3 × 10 min). The cells were immersed in DAPI for nuclear staining and mounted with FluorSave. To check the effect of 1,6‐Hexanediol on disrupting GFP‐NCAP aggregates, cells were untreated or treated with 100 μm arsenite for 1 h. Then, the cells were treated with 5% 1,6‐Hexanediol in PBS for 5 min, fixed and processed for microscopy as described above. To check the effect of RNase A, purified NCAP aggregates were incubated with −/+RNase A for 6 h as described before [40]. To check the effect of mitoxantrone, purified NCAP aggregates were incubated with 1 μm mitoxantrone or vehicle for 24 h as described previously [40]. The untreated or treated samples were placed as a drop in a 3.5 cm glass‐bottom dish for imaging. Imaging was done using a ZEISS LSM 780 laser scanning confocal microscope with zen (blue edition) software. To quantify SGs and colocalization of NCAP with SG proteins, images were captured under 12 different microscopic fields (60× objective) from individual‐stained slides (˜ 10 cells/field). NCAP granules were counted and expressed as a percentage of cells that shows colocalization of NCAP aggregates with the corresponding SG protein compared to total number of cells. For the quantification of droplets and aggregates, images were captured under 12 different microscopic fields (60× objective). The area of aggregates was quantified using imagej software [36, 41].

### Protein pulldown

A549 cells transfected with pcDNA3.1(+)‐N‐GFP‐NCAP‐His (custom made from GenScript, USA) were untreated or treated with arsenite or heat shock. The cells lysates were subjected to pulldown using anti‐His antibodies as described previously [22]. Normal rabbit serum (NRS) was used to control non‐specific binding. The pulldown samples were subjected to immunoblotting using antibodies to NCAP, G3BP1, G3BP2, YTHDF3, USP10 and PKR.

### Phase separation assays

Phase separation assays were conducted as described previously [42, 43]. Briefly, 60 μm GFP‐NCAP or 50 μm RFP‐G3BP1 were mixed with 0.05 μg μL^−1^ of total RNA isolated from arsenite treated A549 cells in a total volume of 20 μL. Then, 10 μL of the sample was transferred as a drop on a 3.5 cm glass‐bottom dish (WillCo‐dish, type 3522, WillCo Wells B.V.). Samples were then observed under a phase‐contrast microscope (Zeiss Axiovision, Zeiss, White Plains, NY, USA) under 20× and 32× objectives. All images were captured within 10 min following LLPS induction. To assess the surface wetting of GFP‐NCAP or RFP‐G3BP1 droplets, the phase‐separated samples were kept at room temperature either in (a) microfuge tube or placed on (b) glass‐bottom dish to monitor surface wetting and incubated for 2 min, 5 min, 10 min and 20 min. Samples were taken from the microfuge tube at regular intervals (2 min, 5 min, 10 min and 20 min), placed on a glass‐bottom dish and immediately imaged (within 1 min). Samples that were kept incubated on the glass slides were also imaged at regular intervals (2 min, 5 min, 10 min and 20 min) and imaged. To check the effect of 1,6‐Hexanediol in dissolving the droplets, the phase‐separated droplets were treated with 10% 1,6‐Hexanediol for 5 min before imaging.

### Time‐lapse microscopy

A549 cells were seeded in 3.5 cm glass‐bottom dishes (WillCo‐dish, type 3522, WillCo Wells B.V.). They were transfected with GFP‐NCAP, RFP‐G3BP1 or both for 24 h. The cultures were placed in a 37 °C chamber equilibrated with humidified air containing 5% CO_2_ while working with video microscopy. Time‐lapse microscopy was performed with a ZEISS LSM 780 microscope using a 60× oil immersion objective. Images were captured using ZEN (blue edition) software every 1 s for 30 min, and the movies were made out of the time‐lapse series using imagej software. For the analysis of droplet dynamics and surface wetting, phase‐separated GFP‐NCAP and RFP‐G3BP1 were placed as a drop in a 3.5 cm glass‐bottom dish. Images were captured every 0.5 s for 5 min or 20 min, and the movies were made as described above.

### NCAP‐mitoxantrone molecular docking

The three‐dimensional structure of RNA‐binding domain (RBD) of NCAP (PDB: 6VYO) of SARS‐CoV‐ 2 was retrieved from the Protein Data Bank (PDB) (https://www.rcsb.org/). The protein structure was subjected to docking using mitoxantrone as the ligand, structure retrieved from PubChem (https://pubchem.ncbi.nlm.nih.gov/), using Cavity‐detection guided Blind Docking (CB‐Dock; http://cao.labshare.cn/cb‐dock/) [44].

### RNA electrophoretic mobility shift assay (EMSA)

RNA EMSA to detect direct binding of NCAP to RNA probe (5’‐UGCUUACGGUUUCGUCCGUGUUGC‐3’), corresponding to 5’UTR leader sequence of SARS‐CoV‐2 (MN908947), was performed using a LightShift Chemiluminescent RNA EMSA kit (Thermo Fisher Scientific) according to the manufacturer’s instructions. In brief, 5 nm biotin tagged probe was incubated with recombinant NCAP in binding buffer (100 mm HEPES, pH 7.3, 200 mm KCl, 10 mm MgCl_2_, 10 mm DTT, 5% glycerol and 2 μg tRNA) in a total volume of 20 μL for 30 min. Control reactions were set up using a 200‐fold molar excess concentration of unlabelled probe along with labelled probe. The reaction products were mixed with 5 μL of 5× loading buffer and subjected to electrophoresis in a 4–6% native polyacrylamide gel in 0.5× TBE buffer (100 V for 8 × 8 × 0.1 cm gel) until the bromophenol blue dye has migrated 3/4 down the length of the gel. The RNA–protein complexes were then transferred to a nylon membrane and cross‐linked for 60 s at 120 mJ cm^−2^ using a commercial UV light cross‐linker (Stratagene) equipped with 254‐nm UV light lamps. The membranes were then developed as described before [22].

### Isolation of NCAP aggregates, RNase A and mitoxantrone treatments

NCAP aggregates were isolated as described previously [40, 45]. Briefly, cells were grown in 10 cm plates to 75–90% confluency, transfected with GFP‐NCAP and stressed with arsenite (100 μm) or heat shock (41 °C) for 1 h. The cells were collected, lysed for 10 min on ice with ice‐cold NP‐40 lysis buffer and centrifuged the lysates at 1000 ***g*** for 5 min at 4 °C to pellet nuclei. Next, the supernatant was collected in a 1.5 mL microfuge tube and centrifuged at 18 000 ***g*** for 20 min at 4 °C to pellet the NCAP aggregates and resuspended in ice‐cold NP‐40 lysis buffer. The resuspended aggregates were further centrifuged at 850 ***g*** for 2 min at 4 °C to pellet membrane‐bound organelles and other large debris. The remaining supernatants were collected as the NCAP‐enriched fractions.

## Results

### SILAC mass spectrometry identification of NCAP‐interacting proteins

**Fig. 1:**
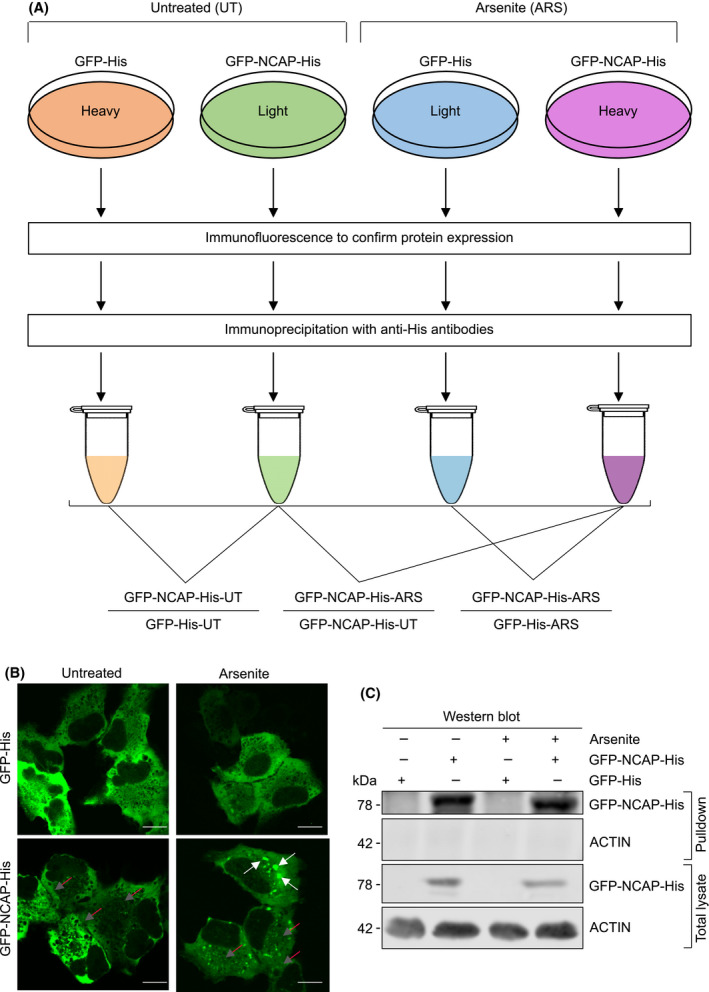
Identification of NCAP‐interacting proteins. (A) Scheme depicting the identification of NCAP‐interacting proteins by immunoprecipitation and SILAC mass spectrometry. (B) Localization of GFP‐NCAP‐His in A549 cells. Note that NCAP is mainly present as diffused in the untreated cells (red arrows) while it is present as both aggregated (white arrows) and diffused (red arrows) in the arsenite‐treated cells. A549 cells transfected with GFP‐His is used as the control. Scale, 10 μm. (C) western blot showing the pulldown of GFP‐NCAP‐His from A549 cells untreated or treated with arsenite. GFP‐His is used as a control in the transfection.

An antibody pulldown approach coupled with SILAC mass spectrometry isolated NCAP interacting proteins in A549 lung cancer cells. GFP was ligated in frame with NCAP‐His to generate a GFP‐NCAP‐His expression plasmid (pcDNA3.1(+)‐N‐GFP‐NCAP‐His). GFP‐NCAP‐His or a GFP‐His control was transiently expressed in A549 cells cultured in SILAC medium containing isotopically stable ‘heavy’ or ‘light’ amino acids (see Methods for more details). Cells were then treated with arsenite (ARS) or untreated (UT) for 1 h to generate four experimental cell populations [GFP‐His‐UT (Heavy), GFP‐NCAP‐His‐UT (Light), GFP‐His‐ARS (Light) and GFP‐NCAP‐His‐ARS (Heavy)], each prepared in triplicate (Fig. 1A). Expression of GFP‐NCAP‐His was confirmed by immunofluorescence (IF; Fig. 1B). GFP‐NCAP‐His showed a predominantly diffuse distribution in the cytoplasm of untreated cells (see red arrows in Fig. 1B; untreated panel). In contrast, a proportion of GFP‐NCAP‐His formed distinct aggregates (see white arrows in Fig. 1B; arsenite panel) in addition to diffused staining (see red arrows in Fig. 1B; arsenite panel). GFP‐His is used as the control in the above experiments.

**Fig. 2:**
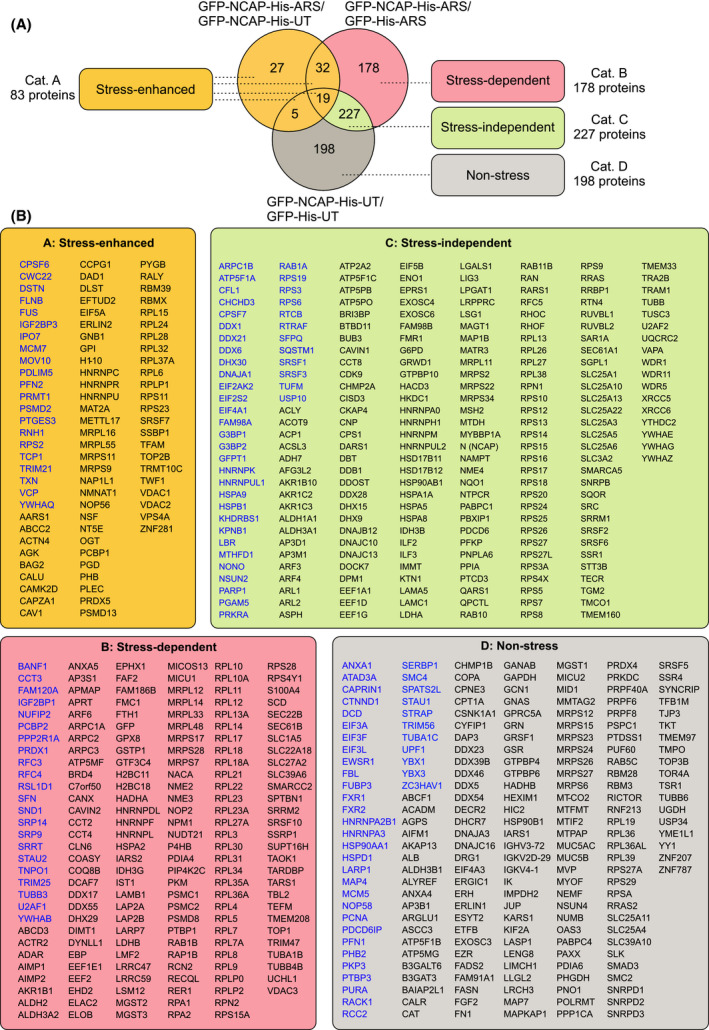
NCAP‐associated proteins. (A) Venn diagram showing comparison and categorization (4 categories) of NCAP‐associated proteins in unstressed and arsenite‐stressed cells (see text for more details). (B) Proteins that come under the above categories are listed in boxes. SG‐interacting proteins already reported in the literature are shown in blue letters

The cells in the above four conditions were harvested and lysed, and subjected to antibody pulldown using anti‐His antibodies (see Methods). The pulldown of NCAP was confirmed by western blotting (Fig. 1C). The pulldown samples were processed for LC‐MS/MS, as described [36]. The mass spectrometry raw data are provided in Table S1. Abundance scores for each protein were averaged from triplicates of three different comparisons, namely GFP‐NCAP‐His‐UT/GFP‐His‐UT, GFP‐NCAP‐His‐ARS/GFP‐His‐ARS and GFP‐NCAP‐His‐ARS/GFP‐NCAP‐His‐UT (Table S2). Statistically significant scores (i.e. *P*‐value < 0.05) were selected, and the three conditions were compared in a Venn diagram (Fig. 2A). Data were organized into four categories of NCAP‐associated proteins after arsenite stress, namely interactions that were stress‐enhanced (yellow shaded), stress‐dependent (red shaded), stress‐independent (green shaded) and non‐stress‐specific (grey shaded) (Fig. 2A). Stress‐enhanced (Category A: 83) is a small group of proteins that interact with NCAP under ambient conditions, and their interaction is enhanced following stress; the stress‐dependent group (Category B: 178 proteins) consists of new protein interactors of NCAP after the stress treatment; the stress‐independent group (Category C: 227 proteins) consists of proteins that interact with NCAP irrespective of stress; and non‐stress‐specific interactors (Category D: 198 proteins) associate with NCAP only under ambient conditions. Proteins included in each of the above categories are listed in Fig. 2B.

### Pathway analysis of NCAP interacting proteins

**Fig. 3:**
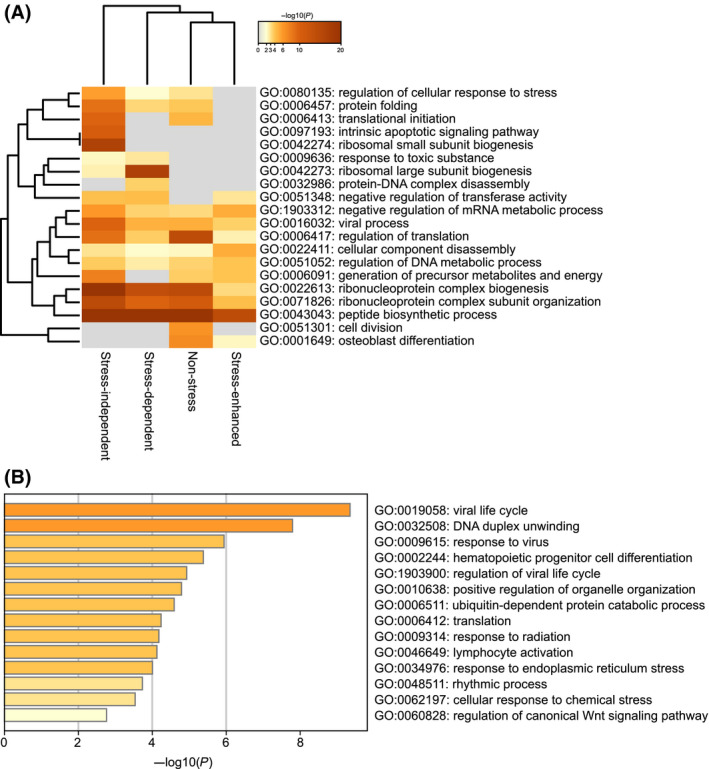
Pathway analysis using Metascape software (http://metascape.org). (A) Pathway analysis using the four categories of proteins (Fig. 2). (B) Pathway analysis of NCAP‐interacting immunoregulatory proteins.

Pathway analysis identified diverse biological functions of NCAP‐interacting proteins, including regulation of cell stress response, protein folding, translational initiation, apoptosis, ribosome biogenesis, viral process, regulation of translation, ribonucleoprotein complex biogenesis, peptide biosynthetic process, cell division and osteoblast differentiation (Fig. 3A). Protein interaction network analyses using STRING (https://string‐db.org) is shown in Fig. [Link], [Link] to demonstrate the connection between different interactors. While comparing the NCAP interacting proteins with the SG database [46], a large set of NCAP interactors were found to be identified previously as SG associated (represented by blue letters in Fig. 2B). Also, several proteins that formed NCAP complexes were involved in immunoregulatory functions. A selected number of those proteins are provided in Table 1. Pathway analysis of NCAP interacting immunoregulatory proteins showed that they were involved in response to virus, regulation of viral life cycle, DNA duplex unwinding, hematopoietic progenitor cell differentiation, translation, lymphocyte activation, different stress responses and Wnt signalling (Fig. 3B).

**Table 1:** A selected number of NCAP interacting proteins involved in immune response and their associated functions

No.	Protein	Functions	Type of interaction	References
1	IMPDH2	Regulates NF‐kB activation and supports SARS‐CoV infection	Non‐stress	[75]
2	TRIM56	Positive regulator of innate immune response.	Non‐stress	[78]
3	ANXA1	Plays a vital role in TLR activation, leading to an augmentation in the type 1 IFN antiviral cytokine response; Promotes RIG‐I‐dependent signalling and apoptosis *via* regulation of the IRF3–IFNAR–STAT1–IFIT1 pathway in A549 lung epithelial cells.	Non‐stress	[80, 81]
4	AP3B1	Required for the production of pro‐inflammatory cytokines in response to viral nucleic acids; significantly enriched in COVID‐19 patients experiencing severe cytokine storms; Crucial for the trafficking of TLR9 to specific endosomal compartments for the induction of type I interferon.	Non‐stress	[82, 83, 84]
5	ASCC3	Inhibits IFN‐signalling to dampen antiviral innate immunity.	Non‐stress	[92]
6	BAIAP2L1	Involved in MAVS degradation, leading to downregulation of antiviral responses.	Non‐stress	[93]
7	HSP90B1	Amplifies innate and adaptive immune responses via interaction with TLR2 and TLR4 ligands.	Non‐stress	[85]
8	PPP1CA	Dephosphorylates RNA sensors, RIG‐I (DDX58) and MDA5 (IFIH1), to induce antiviral IFN‐b production.	Non‐stress	[86]
9	PURA	Regulates several human viruses that replicate in the central nervous system (CNS), including human immunodeficiency virus type I (HIV‐1) and JC virus (JCV).	Non‐stress	[107]
10	RICTOR	Reduces TLR4‐mediated inflammation by regulating the cellular localization of FOXO1.	Non‐stress	[108]
11	RPL19	Facilitates viral multiplication in cells that express TLR3 in endosomes while inhibiting viral multiplication in cells bearing TLR3 on their cell membrane; Interacts with MIF and attenuates its pro‐inflammatory function.	Non‐stress	[109, 110]
12	YBX1	Functions as a porter to lead influenza virus ribonucleoprotein complexes to microtubules; Supports the early and late stages of HIV replication.	Non‐stress	[94, 95]
13	YY1	Negatively regulates TLR3‐induced expression of IFN‐b and acts downstream of TLR3 to limit the level and duration of antiviral response.	Non‐stress	[87]
14	VCP	Involved in the maturation of virus‐loaded endosomes	Stress‐enhanced	[76]
15	EFTUD2	Novel innate immune regulator that restricts Hepatitis C virus infection through an RIG‐I/MDA5‐mediated, JAK‐STAT‐independent pathway.	Stress‐enhanced	[88]
16	PCBP1	Critical in regulating MAVS degradation for both fine‐tuning antiviral immunity and preventing inflammation.	Stress‐enhanced	[89]
17	TRIM21	Negatively regulates the innate immune response to intracellular double‐stranded DNA; Interacts with MAVS to positively regulate innate immunity	Stress‐enhanced	[111, 112]
18	EIF2AK2 (PKR)	An essential mediator of the antiviral and anti‐proliferative actions of interferon (IFN); Recruited to stress granules by G3BP1 to promote innate immune responses at both transcriptional and translational levels.	Stress‐independent	[23, 113]
19	G3BP1	Inhibits RNA virus replication by positively regulating RIG‐I‐mediated cellular antiviral response; Recruits protein kinase R to promote multiple innate immune antiviral responses.	Stress‐independent	[23, 114, 115]
20	HSPA1A	Highly upregulated at the maternal–fetal interface during maternal COVID‐19; Mediates protective antiviral immunity in response to measles virus (MeV) brain infection.	Stress‐independent	[90, 91]
21	LGALS1	Stimulates monocyte migration *via* the p44/42 MAP kinase pathway.	Stress‐independent	[116]
22	NAMPT	Activates Toll‐Like Receptor 4 to Induce NFκB signalling and inflammatory lung injury.	Stress‐independent	[117]
23	PRKRA	Activated by double‐stranded RNA which mediates the effects of interferon in response to viral infection.	Stress‐independent	[118]
24	SQSTM1	Key intracellular target of innate defence regulator‐1 (IDR‐1).	Stress‐independent	[119]
25	XRCC5	Vaccinia virus protein C16 influences the immune response by binding to the XRCC6/XRCC5 (Ku70/80) complex, thus, blocking PRKDC‐dependent DNA sensing in fibroblasts.	Stress‐independent	[120]
26	ADAR	A negative regulator of type I interferon‐mediated signalling pathway.	Stress‐dependent	[96]
27	PCBP2	A negative regulator in MAVS‐mediated antiviral signalling; Synergizes with PCBP1 in MAVS inhibition.	Stress‐dependent	[89, 97]

### NCAP associates with stress granules (SGs) in cells

**Fig. 4:**
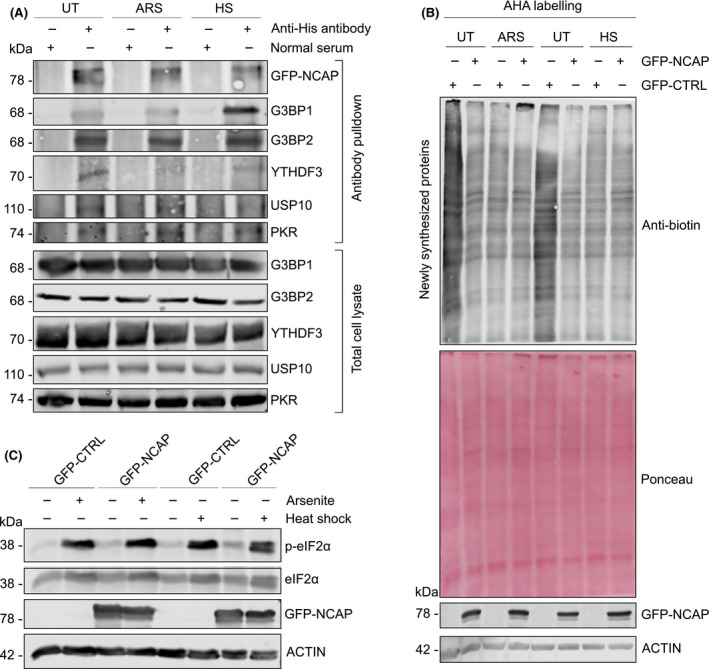
NCAP interacts with SG proteins. (A) Interaction of NCAP with G3BP1, G3BP2, YTHDF3, USP10 and PKR. A549 cells transfected with GFP‐NCAP were unstressed, treated with arsenite or heat shock and subjected to co‐IP using anti‐His antibodies. The pulldown samples and total cell lysates were subjected to western blotting with indicated antibodies. (B) NCAP reduces global protein synthesis in A549 cells as measured by the Click chemistry‐AHA method (see Methods for more details). Ponceau staining and actin were used as loading controls. (C) NCAP does not affect the phosphorylation of eIF2α. A549 cells transfected with GFP‐NCAP were untreated, treated with arsenite or heat shock and subjected to western blotting with antibodies against p‐eIF2α, eIF2α, NCAP and ACTIN.

Since NCAP is associated with SG proteins, a direct interaction of NCAP and SG‐linked factors, including G3BP1, G3BP2, YTHDF3, USP10 and PKR (EIF2AK2) was tested by co‐immunoprecipitation. His‐tagged NCAP was pulldown from untreated or stressed (oxidative or heat shock) cells using anti‐His antibodies and subjected to western blotting using antibodies against G3BP1, G3BP2, YTHDF3, USP10 and PKR. We found that NCAP strongly interacted with the above proteins (Fig. 4A). Similar to our results, G3BP1 and G3BP2 were previously identified as interactors of NCAP that further validates our mass spectrometry data [47, 48]. We found that NCAP expression reduced cellular synthesis of newly translated proteins as revealed by Click‐AHA labelling [35], indicating that global translation is repressed in NCAP expressing cells and that NCAP may impact cellular mRNA translation (Fig. 4B). RNA viruses induce the phosphorylation of eIF2α, which in turn induces the repression of cellular protein synthesis [49, 50]. To assess the effect of NCAP on the repression of protein synthesis is due to eIF2α phosphorylation, we checked p‐eIF2α levels in NCAP expressing cells. We found that eIF2α is not phosphorylated until the cells are treated with arsenite or heat shock (Fig. 4C). This suggests that NCAP might use a different mechanism than other viral proteins to repress protein synthesis. By interacting with the SG‐nucleating protein G3BP1 and repressing global protein synthesis using novel mechanisms, NCAP might regulate mRNA translation and SG formation [6].

**Fig. 5:**
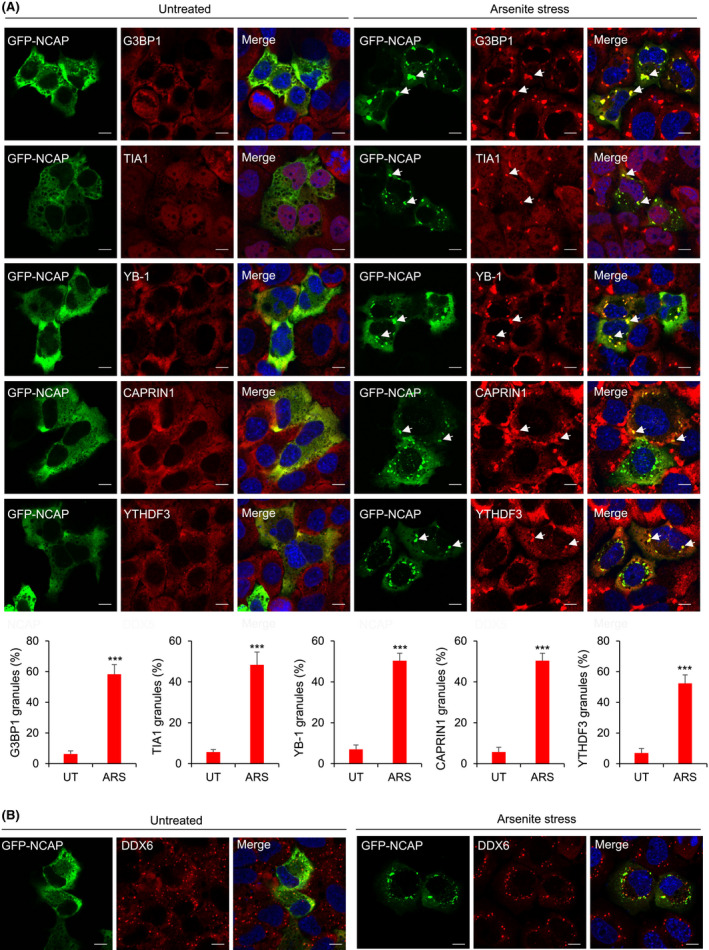
NCAP associates with SGs. (A) A549 cells transfected with GFP‐NCAP were untreated or treated with 100 μm arsenite for 1 h and subjected to IF using antibodies to G3BP1, TIA1, YB‐1, CAPRIN1 and YTHDF3. Note that NCAP colocalizes with all the tested SG proteins. Quantification of colocalization is shown in the bottom panel. Mean values ± SEM are shown for three independent experiments; ***P < 0.001. (B) NCAP granules do not colocalize with PBs. Untreated or arsenite stressed GFP‐NCAP expressing cells were stained with antibodies to PB marker DDX6. Scale, 10 μm.

Nucleocapsid N protein from SARS‐CoV (SARS‐CoV‐1) associates with SGs [51]. To test NCAP’s association with SGs, GFP‐NCAP was transfected into cells and treated with oxidative or heat stress. In untreated cells, GFP‐NCAP was apparent in a diffused form (Fig. 5A, left panels) with only < 5% of transfected cells showing a granular pattern for GFP‐NCAP, and they rarely colocalized with SGs. The non‐induction of SGs by NCAP overexpression in untreated cells suggests that NCAP might not be an SG‐nucleating protein. SG‐nucleating proteins are SG‐associated proteins that induce spontaneous SG formation when overexpressed in the cells without stress treatment (e.g. G3BP1) [52]. We found that GFP‐NCAP could translocate in those SGs formed by the overexpression of G3BP1 in untreated cells, indicating that NCAP can accumulate in SGs formed by SG‐nucleating proteins such as G3BP1 (Fig. [Link], [Link]). After arsenite stress, GFP‐NCAP translocated from the cytosol to SGs and colocalized with SG proteins, including G3BP1, TIA1, YB‐1, CAPRIN1 and YTHDF3 (Fig. 5A, right panels). Heat shock stress also shifted the localization of GFP‐NCAP from the cytosol to SGs (Fig. [Link], [Link]).

We also found that GFP‐NCAP expressing cells formed SGs less efficiently (˜50% of cells) compared to GFP‐CTRL expressing cells (˜90% of cells), indicating that NCAP could inhibit or disrupt SG formation as reported [53, 54] (Fig. [Link], [Link]). NCAP did not show colocalization with PB marker DDX6 in arsenite treatment (Fig. 5B) or heat shock (Fig. [Link], [Link]). NCAP‐PB interactions cannot be completely ruled out as SGs and PBs are dynamically linked mRNP remodelling sites [55]. NCAP aggregates in cells were dynamic, exhibited occasional fusion and fission, and colocalized with G3BP1 [Video [Link], [Link] (GFP‐NCAP aggregates), Video [Link], [Link] (RFP‐G3BP1 aggregates) and Video [Link], [Link] (colocalization of GFP‐NCAP and RFP‐G3BP1 aggregates); Fig. [Link], [Link]A (time‐lapse images of GFP‐NCAP aggregates), and Fig. [Link], [Link]B (time‐lapse images of RFP‐G3BP1 aggregates)]. Together, these results suggest that NCAP can directly interact with SG proteins and associate with SGs in oxidative and heat‐stressed cells.

### Liquid–liquid phase separation of NCAP

**Fig. 6:**
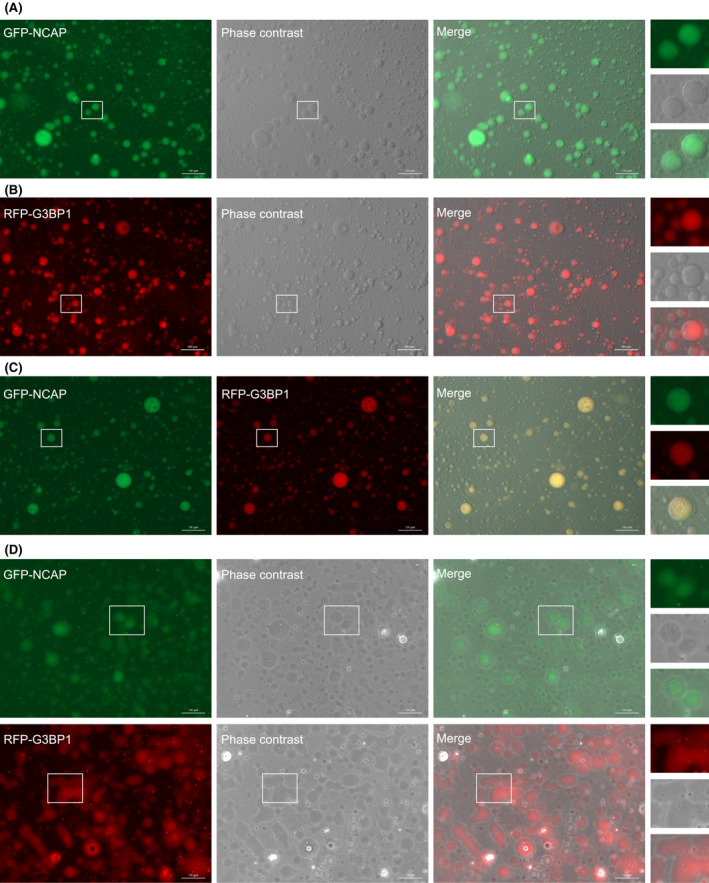
NCAP undergoes LLPS to form liquid droplets. (A) GFP‐NCAP is mixed with RNA to induce phase separation. The images were captured in a fluorescence microscope under 20× objective. (B) RFP‐G3BP1 is similarly phase separated with RNA. (C) GFP‐NCAP and RFP‐G3BP1 droplets were mixed and imaged. Note that droplets formed by both GFP‐NCAP and RFP‐G3BP1 colocalize. (D) GFP‐NCAP or RFP‐G3BP1 has liquid‐like properties. Droplets formed by GFP‐NCAP or RFP‐G3BP1 were allowed to stay on the surface for > 20 min and imaged. Note that the droplets flattened with time and wetted the surface showing their liquid property. A part of the image is enlarged and displayed on the right side of respective panels. Scale, 100 μm.

SG‐associated proteins such as G3BP1, FUS, YTHDF3 and TDP‐43 are intrinsically disordered proteins (IDPs) with an enhanced capacity to undergo liquid–liquid phase separation (LLPS) to form liquid droplets [56]. Phase separation property of these proteins alone or in association with additional RNA‐binding proteins (RBPs) or RNAs modulate the formation of SGs in cells [42, 43, 57]. NCAP interaction with G3BP1 and further association with SGs suggest that the NCAP can undergo phase separation to form liquid droplets. Protein disorder prediction using IUPred2A [58] indicates that NCAP is an intrinsically disordered protein that further supports this hypothesis [59, 60] (Fig. [Link], [Link]A). To characterize its role in phase separation, NCAP was cloned in fusion with GFP, and the purified recombinant protein was used for phase separation studies. NCAP phase‐separated to form droplets when the purified GFP‐NCAP was incubated with total RNA isolated from A549 cells (Fig. 6A). G3BP1, an intrinsically disordered and SG nucleating factor (Fig. [Link], [Link]B), was used as a control in the phase separation studies [52]. Recently several research groups observed RNA‐dependent phase separation of G3BP1 [42, 43, 57]. When G3BP1 was cloned in fusion with RFP and purified, the recombinant RFP‐G3BP1 also underwent phase separation with RNA to form liquid droplets (Fig. 6B). Mixing of GFP‐NCAP and RFP‐G3BP1 droplets demonstrated colocalization in multiple droplets (Fig. 6C).

Time‐lapse images and movies of GFP‐NCAP and RFP‐G3BP1 droplets illustrated that both types of droplets were dynamic and showed occasional fusion and fission properties [Video [Link], [Link] (GFP‐NCAP droplets), Video [Link], [Link] (RFP‐G3BP1 droplets), Video [Link], [Link] (Colocalization of GFP‐NCAP and RFP‐G3BP1 droplets), Video [Link], [Link] (GFP‐NCAP droplets under phase contrast), Video [Link], [Link] (RFP‐G3BP1 droplets under phase contrast), Fig. [Link], [Link]A (time‐lapse images of GFP‐NCAP droplets) and Fig. [Link], [Link]B (time‐lapse images of RFP‐G3BP1 droplets). Together, these results indicate that GFP‐NCAP can undergo LLPS with RNA to form dynamic liquid droplets, and these droplets can associate with liquid droplets formed by G3BP1.

To assess the liquid‐like properties of GFP‐NCAP and RFP‐G3BP1, we have incubated the phase‐separated droplets either in a microfuge tube or on the surface of a glass‐bottom dish and imaged them at regular intervals. We noticed that both GFP‐NCAP and RFP‐G3BP1 droplets incubated in solution in the microfuge tube remained in the droplet form (> 20 min). On the other hand, the droplets that were in touch with the glass surface for a prolonged time (> 20 min) wetted the surface, suggesting that GFP‐NCAP (Fig. 6D, top panel; Fig. [Link], [Link]A) and RFP‐G3BP1 (Fig. 6D, bottom panel; Fig. [Link], [Link]B) droplets can have liquid‐like properties. We also monitored the surface wetting of GFP‐NCAP and RFP‐G3BP1 using time‐lapse movies [Video [Link], [Link] (surface wetting of GFP‐NCAP), Video S10 (surface wetting of RFP‐G3BP1) and Video S11 (colocalization of GFP‐NCAP and RFP‐G3BP1)]. To further characterize the liquid‐like properties of NCAP, we treated the GFP‐NCAP expressing cells, or *in vitro* GFP‐NCAP droplets with 1,6‐Hexanediol, a compound used to dissolve liquid–liquid phase‐separated condensates [61]. Treatment with 1,6‐Hexanediol disrupted both GFP‐NCAP *in vitro* droplets (Fig. [Link], [Link]A) and cellular aggregates (Fig. [Link], [Link]B). G3BP1 was used as a control in these experiments. Like GFP‐NCAP, RFP‐G3BP1 *in vitro* droplets (Fig. [Link], [Link]A) and cellular aggregates (Fig. [Link], [Link]B) were also disrupted by treatment with 1,6‐Hexanediol. Together, these results suggest that the condensates formed by NCAP and G3BP1 can have liquid‐like properties.

### NCAP requires RNA for phase separation and SG association

**Fig. 7:**
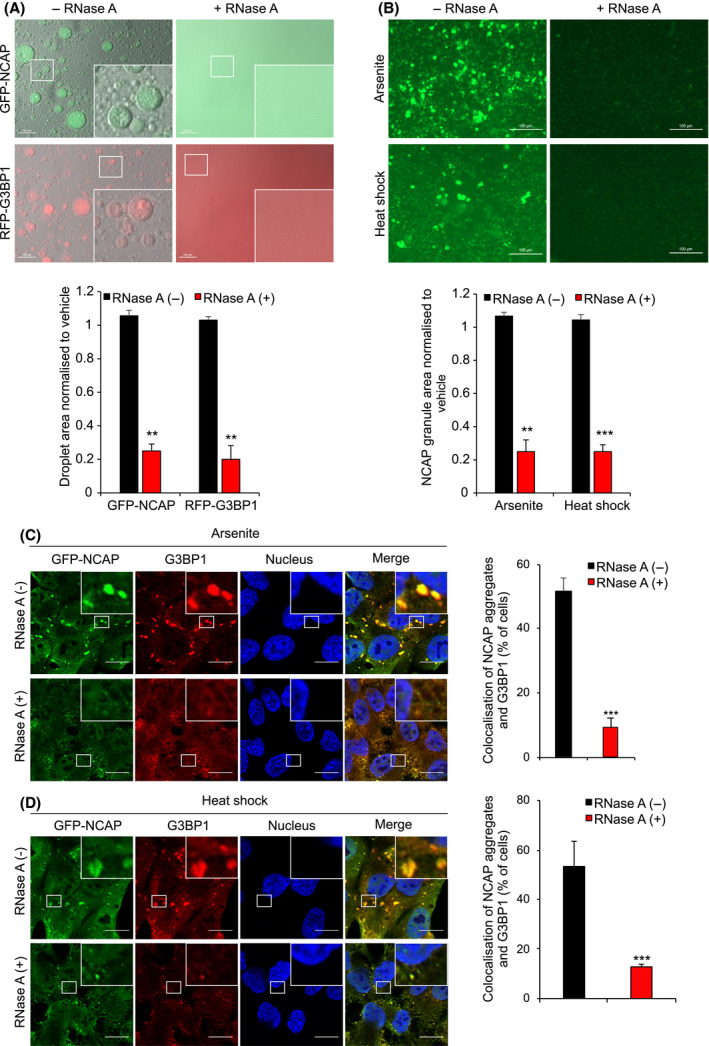
Phase separation and SG association of NCAP requires RNA. (A) GFP‐NCAP or RFP‐G3BP1 droplets phase separated with RNA were incubated in presence or absence of RNase A. Note that RNase A treatment dissolved both GFP‐NCAP and RFP‐G3BP1 droplets. Scale, 100 μm. The bottom panel shows the quantification of phase separation. (B) GFP‐NCAP expressing cells were treated with 100 μm arsenite or heat‐shocked at 41 °C for 1 h. GFP‐NCAP granules were isolated from the cells and incubated with or without RNase A for 6 h. The samples were observed under a fluorescence microscope. Note that arsenite treatment‐ or heat‐shock‐induced granules were significantly dissolved in the presence of RNase A, confirming the requirement of RNA for the integrity of NCAP granules. Scale, 100 μm. The bottom panel shows the quantification. (C‐D) GFP‐NCAP expressing A549 cells were untreated or treated with 100 μm arsenite (C) or heat‐shocked at 41 °C (D) for 50 min. The cells were gently permeabilized with detergent, and the live cells were untreated or treated with RNase A for 10 min. The fixed cells were subjected to IF using anti‐G3BP1 antibodies. Note that treatment with RNase A significantly reduced the accumulation of GFP‐NCAP in SGs. Scale, 10 μm. The right‐side panels show quantification of GFP‐NCAP association with SGs. Mean values ± SEM are shown for three independent experiments; **P < 0.01, ***P < 0.001.

RNA is a critical factor in the phase separation of different liquid condensates [62, 63, 64, 65]. For assessing the requirement of RNA in NCAP phase separation, GFP‐NCAP droplets (see the section on LLPS of NCAP) were mixed with RNase A or vehicle and imaged (Fig. 7A). Treatment with RNase A immediately dissolved the GFP‐NCAP droplets compared to vehicle‐treated droplets, indicating that phase separation of NCAP requires RNA. RNase A treatment experiments used G3BP1 as a control (Fig. 7A). Previous studies confirm the requirement of RNA for phase separation of G3BP1 [42, 43, 57]. As expected, similar to NCAP, G3BP1 droplets were also dissolved by RNase A treatment, confirming the requirement of RNA in G3BP1 droplet formation (Fig. 7A).

The RNA requirement for NCAP association with SGs was tested further by *in vitro* and in cell experiments. NCAP aggregates were purified from arsenite or heat‐stressed GFP‐NCAP‐transfected cells and incubated with RNase A or vehicle for 6 h (see Methods for details) [66]. Treatment with RNase A significantly reduced GFP‐NCAP aggregates from arsenite and heat‐stressed cells, compared to vehicle‐treated controls (Fig. 7B), indicating that *in vitro* NCAP aggregates require RNA for their integrity. To determine RNA‐dependent association of NCAP with SGs, arsenite or heat‐stressed GFP‐NCAP cells were gently permeabilized with detergent and treated with RNase A (see Methods for details). The cells were fixed and subjected to IF with antibodies to G3BP1. RNase A treatment significantly reduced GFP‐NCAP association with SGs in arsenite (Fig. 7C) or heat‐stressed (Fig. 7D) cells compared to vehicle‐treated cells. Together, these results suggest that RNA plays a critical stimulatory role in the *in vitro* assembly and SG association of NCAP.

### Mitoxantrone inhibits NCAP assembly

**Fig. 8:**
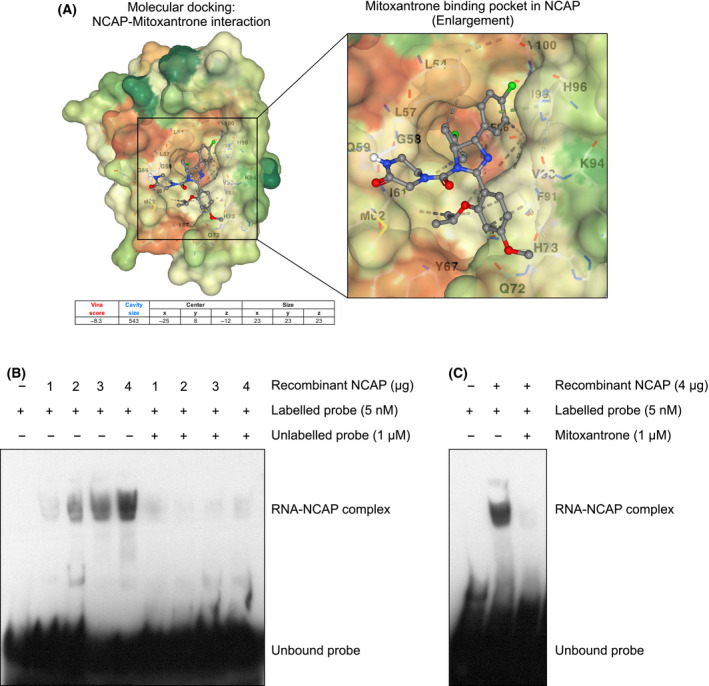
Mitoxantrone disrupts RNA‐NCAP interaction. (A) Docking studies illustrating the interaction of mitoxantrone with RBD‐NCAP (see Methods for more details). (B) RNA EMSA analysis to determine the direct binding of NCAP to the RNA. Biotin‐labelled RNA probe (24 bp) corresponding 5’ leader sequence of SARS‐CoV‐2 genome is mixed with increasing concentration of recombinant NCAP and subjected to EMSA. A probe mobility shift in the presence of NCAP is indicated. A 200‐fold molar excess concentration of unlabelled RNA was added along with labelled probe to demonstrate the specificity of NCAP‐RNA complex formation. (C) EMSA analysis shows that treatment with mitoxantrone disrupted NCAP‐RNA complex formation (see Methods for more details).

Since RNA is critical for NCAP to form aggregates, an RNA intercalating agent, mitoxantrone, was tested for its ability to inhibit NCAP functions. Mitoxantrone is an antineoplastic agent approved for treating several cancers and exhibits antiviral properties [67, 68, 69]. A recent docking study found mitoxantrone‐SARS‐CoV‐2 M protein interactions [70], suggesting that the compound might inhibit the functions of M protein. We have conducted Cavity‐detection guided Blind Docking (CB‐Dock) to test the interaction of NCAP with mitoxantrone (Fig. 8A). We used the NCAP structure corresponding to the RNA‐binding domain (RBD domain; PDB: 6VYO) and mitoxantrone structure derived from PubChem for molecular docking. Docking studies showed that mitoxantrone could insert into a pocket in the NCAP protein structure (Fig. 8A). Interaction of NCAP‐RBD with mitoxantrone suggests that the compound might interfere with NCAP’s RNA‐binding functions. We have tested RNA‐NCAP interaction and its inhibition by mitoxantrone by RNA electrophoretic mobility shift assay (EMSA) as described before [22]. Recombinant NCAP forms a complex with biotin‐tagged RNA probe, corresponding to a part of 5’UTR leader sequences of SARS‐CoV‐2 genome (MN908947) (Fig. 8B). The intensity of the NCAP‐RNA probe complex increased after titrating with increasing concentration of NCAP, and incubation with a higher concentration of unlabelled competitor probe reduced the interaction, showing NCAP‐RNA‐binding specificity (Fig. 8B). Interestingly, including mitoxantrone in the assay mixture reduced the NCAP‐RNA interaction (Fig. 8C). These results illustrate the NCAP‐RNA interaction and the competency of mitoxantrone to disrupt that interaction.

**Fig. 9:**
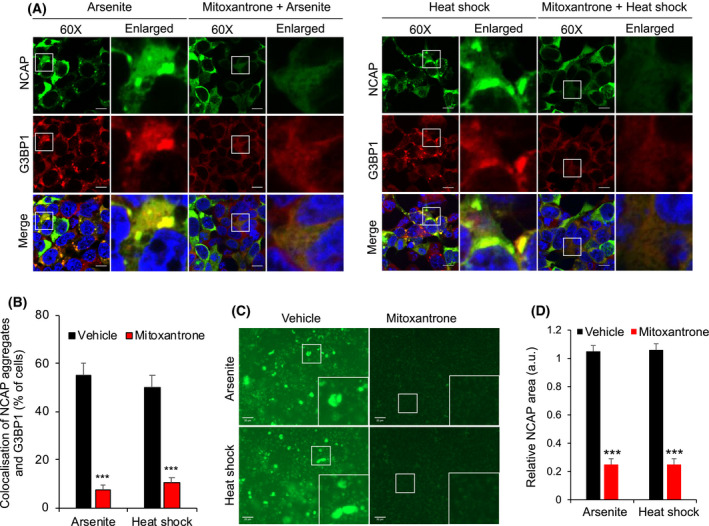
Mitoxantrone inhibits the assembly of NCAP. (A) Mitoxantrone treatment inhibits NCAP assembly in A549 cells. A549 cells expressing GFP‐NCAP were untreated or treated with 1 μm mitoxantrone for 6 h. The cells were then arsenite‐stressed (100 μm) or heat‐shocked (41 °C) for 1 h, and subjected to IF using anti‐G3BP1 antibodies. Note that mitoxantrone treatment significantly reduced the assembly of GFP‐NCAP in SGs in arsenite‐stressed and heat‐shocked cells. Scale, 10 μm. The quantification of cells with GFP‐NCAP in SGs is shown on the bottom left‐side panel (B). (C) Mitoxantrone treatment dissolved *in vitro* purified GFP‐NCAP granules. GFP‐NCAP granules isolated from arsenite‐ or heat‐stressed cells were incubated with or without 1 μm mitoxantrone for 24 h. The samples were imaged under a fluorescence microscope. Note that treatment with mitoxantrone dissolved the GFP‐NCAP granules, as evident from reduced fluorescence. Scale, 20 μm. The right‐side panel shows quantification (D). Mean values ± SEM are shown for three independent experiments; ***P < 0.001.

We also found that mitoxantrone treatment significantly reduced NCAPs translocation to SGs in response to arsenite or heat stress at concentrations as low as 1 μm (Fig. 9A and B; Fig. [Link], [Link]A). Mitoxantrone did not affect protein levels of NCAP or G3BP1, excluding this as a cause for reduced NCAP assembly in SGs (Fig. [Link], [Link]B). Mitoxantrone also disrupted *in vitro* purified NCAP aggregates from arsenite or heat‐stressed cells (Fig. 9C and D). Together, these results highlight the ability of an RNA chelator like mitoxantrone to disrupt NCAP assembly.

## Discussion

This study investigated the interactors of SARS‐CoV‐2 NCAP protein in a human lung cancer cell line. NCAP interacted with hundreds of proteins in unstressed and oxidatively stressed cells. A significant number of NCAP interacting proteins were involved in SG regulation and immune response. Validation of the mass spectrometry data confirmed the interaction of NCAP with G3BP1, G3BP2, YTHDF3, USP10 and PKR. Subsequent studies found that NCAP can assemble in SGs and colocalized with SG proteins G3BP1, TIA1, YB‐1, CAPRIN1 and YTHDF3, in response to stress. Biophysically, NCAP also exhibited liquid–liquid phase separation (LLPS) to form droplets. RNA was an essential determinant of NCAP assembly in SGs in cells and *in vitro* LLPS to form liquid droplets. Mitoxantrone inhibited NCAP assembly in SGs and disrupted *in vitro* purified NCAP aggregates. This study provides insights into NCAP’s biological functions and biophysical phase separation properties and how to interrupt this process.

We found that NCAP forms a complex with several SG components that are mainly RNA‐binding proteins (RBPs). Viral infections can result in transient induction of SGs, complete inhibition of SGs, or alternate assembly and disassembly of SGs during infection [71]. Many reports suggest SG formation as an antiviral response mechanism [72]. The association of NCAP with SGs can modulate viral mRNA translation – potentially sequester viral mRNAs in SGs *via* interaction with NCAP and other SG‐associated RBPs and reduce their translation. A recent multi‐omics approach uncovered direct interaction of viral mRNA with several cellular RBPs and six viral proteins [73]. Thus, SARS‐CoV‐2 infection could remodel the cellular RNA‐bound proteome with wide‐ranging effects on RNA metabolic pathways [73]. As part of promoting the innate immune response, the SG nucleator G3BP1 recruits the stress‐responsive PKR kinase to the SGs, activating it in association with CAPRIN1 [13, 23, 24]. The activated PKR phosphorylates eIF2α to reduce viral mRNA translation and limit viral replication. Supporting this, we found that NCAP interacts with G3BP1 and PKR in unstressed and stressed cells. Also, transfection with NCAP strongly reduced the global mRNA translation measured by the newly synthesized proteins. At the same time, it is notable that we do not see phosphorylation of eIF2α or the formation of SGs in NCAP‐transfected cells without stress with such robust inhibition of translation. Also, the number cells that formed SGs in response to arsenite treatment were lesser in GFP‐NCAP‐transfected cells compared to GFP‐CTRL‐transfected cells, suggesting that NCAP might perhaps prevent the assembly of SGs until additional stress is applied or that NCAP expressing cells require phosphorylation of eIF2α to form SGs. In that case, NCAP might exploit a different mechanism of translational repression and modulation of SG formation than other viral proteins. NCAP inhibits SARS‐CoV‐2 viral RNA‐induced PKR activation, which in turn blocks eIF2α phosphorylation, leading to the impairment of SG formation [53]. Furthermore, SARS‐CoV‐2 infection leads to a global reduction in translation through accelerated degradation of cellular mRNAs, but not of viral mRNAs [74]. This mechanism provides an opportunity for viral mRNA to dominate the mRNA pool in the infected cells and divert the translation machinery in favour of viral mRNA translation. Additionally, the nuclear mRNA export of transcripts induced in response to infection, including innate immune genes, were blocked, leading to the inhibition of their mRNA translation and protein synthesis [74]. Together, these observations suggest that the cells might activate an innate immune response to NCAP expression, which leads to suppression of protein synthesis *via* novel mechanisms of mRNA translation to affect viral replication.

Interestingly, many immunoregulatory factors, including proteins involved in SARS‐CoV‐2 infection, were identified as NCAP interactors in our study (Table 1). A selected number of these proteins include IMPDH2 (regulates NF‐κB activation and supports SARS‐CoV infection) [75], VCP (involved in the maturation of virus‐loaded endosomes) [76], TRIM56 (direct antiviral actions against positive‐sense single‐stranded RNA viruses and positive regulator of innate immune response) [77, 78]), ANXA1 (suppression of inflammation by limiting the production of neutrophil and pro‐inflammatory cytokines) [79, 80, 81], AP3B1 (significantly enriched in COVID‐19 patients experiencing severe cytokine storms) [82, 83, 84], HSP90B1 (TLR signalling) [85], PPP1CA (antiviral IFNB production) [86], YY1 (interacts with STAT1 and activates of IFN‐1 signalling) [87], EFTUD2 (immune regulator that restricts viral infection) [88], PCBP1 (regulates MAVS degradation and fine‐tune antiviral immunity) [89], HSPA1A (protective antiviral immunity and highly upregulated at the maternal–fetal interface during maternal COVID‐19) [90, 91], PRKRA (governs the effects of IFN in response to viral infection), SQSTM1 (key intracellular target of innate defence regulator‐1 (RIG‐1), ASCC3 (inhibits IFN signalling) [92], BAIP2L1 (MAVS degradation leading to downregulation of antiviral response) [93], YBX1 (YB‐1) (supports viral replication) [94, 95], ADAR (a negative regulator of type 1 interferon‐mediated signalling) [96] and PCBP2 (a negative regulator of MAVS‐mediated antiviral signalling) [89, 97]. The inter‐relationship between different NCAP interactors and regulation of associated pathways in response to an actual infection with the SARS‐CoV‐2 virus is likely complex, with the victor (virus or cell) decided by factors including NCAP expression level, viral load, the interplay between the positive and negative immunoregulators, impact on protein translation machinery and physiological state of the cell.

The LLPS of NCAP and its association in SGs might be part of the virus’s life cycle. We speculate that the LLPS of viral proteins can be advantageous for the survival of the virus in different ways. The phase‐separation of viral proteins provides flexibility to recruit and enrich viral proteins and viral transcripts locally, thereby exchanging molecules with host translation machinery for viral mRNA translation or virion assembly. Recent research supports the idea of phase separation of intrinsically disordered viral proteins promoting the formation of liquid condensates. Vesicular stomatitis virus (VSV) infection stimulates liquid‐like condensates using viral proteins involved in replication [98]. Phase separation of N and P proteins of measles virus induces liquid condensates containing the viral genome [99]. Rabies virus infection forms liquid‐like inclusions called Negri bodies (NBs) in the cytoplasm, driven by an IDR in the viral P protein, promoting viral genome replication [30]. NBs release viral nucleocapsids and distribute them to other parts of the cell to form new virions or secondary viral factories. SGs localize close to the NBs. The close association of viral factories with SGs suggests that the viruses may co‐opt SG proteins, including G3BP1, to support replication or virion assembly [12, 30, 100].

Its protein and RNA composition largely influence the LLPS behaviour of SGs [62, 101]. So, it is reasonable to think that SARS‐CoV‐2 may hijack SGs by directly integrating the intrinsically disordered NCAP, which has a capacity for phase separation, into the SGs to enable the viral protein with considerable effect on the phase transition behaviour of SGs. NCAP can then fine‐tune the SGs to phase separate a distinct set of proteins and RNAs advantageous for viral replication. Additionally, phase separation of NCAP could protect NCAP‐interacting viral transcripts during the initial stages of infection and reverse SG assembly using its dynamic liquid‐like property. Sequestered transcripts can be released and shuttled back to viral factories for translation and virion assembly. SG dissociation could also support the viral life cycle by using components in SGs, like initiation factors and RBPs, for stability and translation of viral proteins. A recent study revealed that the SARS‐CoV‐2 nucleocapsid protein phase separated with G3BPs to disassemble SGs and facilitate viral production [54]. Thus, NCAP phase separation, which can also be influenced by post‐translational modifications such as phosphorylation and glycosylation and integration in SGs could provide dominant control over SG function, by changing content of SGs or compromising activation of proteins like PKR, key for innate antiviral immunity [102, 103, 104, 105].

We found that NCAP assembly occurs in the presence of RNA. RNase A degradation or mitoxantrone intercalation of RNA disassembles *in vitro* purified NCAP granules and inhibits NCAP assembly in SGs. Mitoxantrone has been reported to modulate SGs formation, reducing RNA‐dependent recruitment of TDP‐43 to SGs, and preventing subsequent TDP‐43 aggregation in long‐term stressed cells [40]. Besides being a well‐known antineoplastic agent, mitoxantrone also has antiviral properties, suppressing cowpox, monkeypox and herpes simplex virus infections [67, 68, 69]. The current study suggests that mitoxantrone can inhibit NCAP assembly, possibly through interfering NCAPs interaction with RNA.

In conclusion, this work identified interactors of NCAP in unstressed and oxidatively stressed cells, which included components of the SG machinery and immunoregulators. NCAP interacted with SG proteins and assembled in SGs after oxidative and heat‐stress, and its expression reduces global cellular protein translation. NCAP undergoes RNA‐dependent LLPS to form droplets, a mechanism that may enable hijacking host cell translation systems for viral replication and survival. Reducing NCAP function would be disadvantageous for the virus to survive and is a rationale anti‐COVID therapeutic target. Mitoxantrone or other planar moiety containing compounds could be potentially useful to disrupt NCAP assembly and its associated functions. The current study only looked at NCAP’s *in vitro* and cell properties without modelling viral infection as an experimental system in the investigation. We will see more insights on NCAP’s biological processes during viral infection when a detailed study using the SARS‐CoV‐2 virus will be employed.

## Author contributions

SPS conceived the idea, designed and conducted experiments, discussed and analysed the data, and wrote the manuscript. MG discussed and edited the manuscript.

## Supporting information


**Fig. S1**. Protein interaction network analysis of NCAP interacting proteins using string software (https://string‐db.org).

**Fig S2**. Overexpression of G3BP1 induces the translocation of GFP‐NCAP to SGs.

**Fig S3**. NCAP assembles in SGs in response to heat stress.

**Fig S4**. Formation of SGs in GFP‐CTRL expressing cells and their comparison to GFP‐NCAP expressing cells. Scale, 10 μm.

**Fig S5**. NCAP granules do not colocalize with PBs.

**Fig S6**. Dynamic nature GFP‐NCAP and RFP‐G3BP1 granules in cells in response to arsenite treatment.

**Fig S7**. Prediction of disordered regions in (A) NCAP and (B) G3BP1 using IUPred2 (https://iupred2a.elte.hu/).

**Fig S8**. Dynamic nature GFP‐NCAP and RFP‐G3BP1 droplets.

**Fig S9**. Surface wetting of (A) GFP‐NCAP and (B) RFP‐G3BP1 droplets with prolonged incubation (see Methods for more details). Scale, 20 μm.

**Fig S10**. NCAP granules exhibit liquid‐like properties.

**Fig S11**. Mitoxantrone (MXN) treatment inhibits the assembly of GFP‐NCAP in A549 cells.

**Video S1**. GFP‐NCAP association with SGs in arsenite‐stressed cells.

**Video S2**. RFP‐G3BP1 association with SGs in arsenite‐stressed cells.

**Video S3**. Colocalization of GFP‐NCAP and RFP‐G3BP1 in arsenite‐stressed cells.

**Video S4**. GFP‐NCAP droplets formed by phase separation.

**Video S5**. RFP‐G3BP1 droplets formed by phase separation.

**Video S6**. Colocalization of GFP‐NCAP and RFP‐G3BP1 droplets formed by phase separation.

**Video S7**. GFP‐NCAP droplets formed by phase separation (under phase contrast).

**Video S8**. RFP‐G3BP1 droplets formed by phase separation (under phase contrast).

**Video S9**. GFP‐NCAP droplets undergoing surface wetting.

**Video S10**. RFP‐G3BP1 droplets undergoing surface wetting.

**Video S11**. Colocalization of GFP‐NCAP and RFP‐G3BP1 droplets that are undergoing surface wetting.

**Table S1**. List of NCAP interacting proteins in unstressed and arsenite‐stressed A549 cells – Raw data.

**Table S2**. List of NCAP interacting proteins (GFP‐NCAP‐His‐UT/GFP‐His‐UT, GFP‐NCAP‐His‐ARS/GFP‐His‐ARS and GFP‐NCAP‐His‐ARS/GFP‐NCAP‐His‐UT).

## Data Availability

The mass spectrometry proteomics data have been deposited to the ProteomeXchange Consortium *via* the PRIDE [106] partner repository with the data set identifier PXD023989.
